# Sexual Dysfunctions and Gynecomastia in Male Rheumatological Patients Treated with Methotrexate: A Systematic Review

**DOI:** 10.3390/jcm13216455

**Published:** 2024-10-28

**Authors:** Luigi Napolitano, Marco Abate, Francesco Di Bello, Simone Morra, Luigi Cirillo, Giovanni Maria Fusco, Gianluigi Califano, Claudia Collà Ruvolo, Massimiliano Creta, Roberto La Rocca, Felice Crocetto, Biagio Barone, Ilenia Pantano, Pierluigi Russo, Davide Arcaniolo, Celeste Manfredi

**Affiliations:** 1 Department of Neurosciences, Reproductive Sciences and Odontostomatology, University of Naples “Federico II”, 80138 Napoli, Italy; dr.luiginapolitano@gmail.com (L.N.); marcoabate5@gmail.com (M.A.); simonemorra@outlook.com (S.M.); cirilloluigi22@gmail.com (L.C.); giom.fusco@gmail.com (G.M.F.); gianl.califano2@gmail.com (G.C.); c.collaruvolo@gmail.com (C.C.R.); max.creta@gmail.com (M.C.); roberto.larocca@unina.it (R.L.R.); felice.crocetto@gmail.com (F.C.); biagio.barone@aslnapoli1centro.it (B.B.); 2 Rheumatology Unit, Department of Precision Medicine, University Della Campania L. Vanvitelli, 80138 Naples, Italy; ileniapantano@gmail.com; 3 Department of Urology, Fondazione Policlinico Universitario Agostino Gemelli IRCCS, Università Cattolica del Sacro Cuore, Largo Francesco Vito 1, 00168 Rome, Italy; pierluigi92.russo@gmail.com; 4 Urology Unit, Fondazione Policlinico A. Gemelli IRCCS, Largo Agostino Gemelli 8, 00168 Rome, Italy; 5 Urology Unit, Department of Woman Child and of General and Specialist Surgery, University of Campania “Luigi Vanvitelli”, 80138 Naples, Italy; davide.arcaniolo@unicampania.it (D.A.); celeste.manfredi@unicampania.it (C.M.)

**Keywords:** erectile dysfunction, urology, rheumatology, hormones

## Abstract

**Objectives:** The aim of the current review was to elucidate the clinical context and presentation of sexual dysfunction (SD) and gynecomastia in rheumatological patients undergoing methotrexate treatment. Moreover, we aimed also to make physicians aware of the occurrence of these side effects, to adequately inform the patient before starting treatment. **Methods**: Systematic review (PROSPERO id: CRD42022358275) was performed according to preferred reporting items for systematic reviews and meta-analyses. Studies (1 January 1995 to 31 May 2022) were identified by highly sensitive searches of electronic databases (Medline, Embase, Cochrane Library databases). Key terms included: ((“sexual dysfunction” OR “erectile dysfunction” OR “impotence”) AND (“methotrexate” OR “MTX”)) and ((“gynecomastia” OR “male breast”) AND (“methotrexate” OR “MTX”)). **Results**: A total of seven papers (seven case reports), involving a total of eleven patients (min one, max three), were included in the final analysis. The age of the patients ranged from 19 to 68 years (median: 50.9 years). Rheumatoid arthritis (RA) was the most frequent rheumatic disease reported (n = 8, 72.7%). No patients reported erectile dysfunction (ED) and/or gynecomastia before using MTX. Time to onset of SD and gynecomastia after MTX ranged from 2 to 104 weeks (median: 22.7 weeks). ED (n = 5, 45.4%) and gynecomastia (n = 3, 27.2%) were the most common forms of presentation. **Conclusions**: Future prospective controlled studies with a large sample size and long follow-up as well as randomized controlled trials are needed to confirm this association, investigate its pathophysiological basis, assess the safest dosages, evaluate the most appropriate management, and provide clear recommendations.

## 1. Introduction

Male sexual health can be compromised by several factors, including systemic diseases and drugs [1,2,3,4,5]. Erectile dysfunction (ED) shows a global prevalence ranging from 3% to 76.5% and is associated with increasing age, representing an important health problem worldwide [1,3,6,7]. Particularly, ED is defined as “the inability to achieve or maintain an erection sufficient for satisfactory sexual performance with high impact on the quality of life” [6,7]. Gynecomastia, defined as benign enlargement of a male breast due to the proliferation of glandular tissue, is a further condition that can negatively impact the quality of life of men, and it is generally associated with hormonal impairments related with systemic diseases or medications [1]. Both sexual dysfunction (SD) and gynecomastia could be drug-related effects caused by chronic treatments and chemotherapy agents, as previously assessed [8,9,10].

Methotrexate (MTX) is a folate antimetabolite which irreversibly binds and inhibits dihydrofolate reductase (DHFR) [10,11]. MTX reduces metabolically active intracellular folates, decreasing the de novo synthesis of purines and pyrimidines (precursors of DNA and RNA) required for cellular proliferation [10,11]. Furthermore, MTX inhibits the production of cytokines induced by T-cell activation such as IL-4, IL-13, IFN-γ, and TNF-α [10]. Since the 1980s, MTX has been widely used as an immunosuppressive drug in various autoimmune diseases and as an antiproliferative drug in different cancers due to its immunosuppressant and anti-inflammatory properties [10]. Although MTX is well tolerated, many patients report different side effects: nausea, fatigue, dizziness, stomatitis, abdominal pain, hepatic toxicity, pulmonary toxicity, nephrotoxicity, and myelosuppression [10]. Over time, many authors have also reported ED, loss of libido, and gynecomastia as rare adverse effects [3,9,10,12,13,14,15,16]. Generally, most side effects are resolved by lowering the dose or, if needed, discontinuing the drug, although this may promote a flare of the disease [10,15,16]. Although the association between MTX and SD or gynecomastia has been reported in the literature, the evidence on the topic is still very limited [9,14,17,18,19,20]. Boussaid et al., in their systematic review of 25 studies published between 1981 and 2018 and including 27 rheumatological patients, assessed the existence of sexual dysfunction in rheumatological patients, namely hormones imbalances or impairment of sperm quality [21]. Moreover, they expanded on the paucity of data regarding male-related fertility, and, consequently, the lack of guidance on the official guidelines addressing this specific group of patients [21].

The aim of this systematic review was to describe the clinical context and presentation of SD and gynecomastia in rheumatological patients treated with MTX. In addition, we discussed the possible pathophysiological mechanisms underlying these side effects and their management.

## 2. Materials and Methods

### 2.1. Literature Search

**Figure 1:**
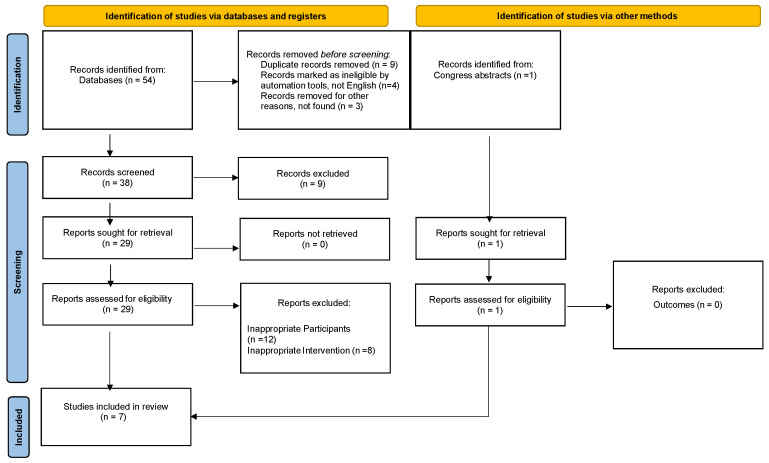
PRISMA diagram depicting inclusion and exclusion criteria of 15 studies focused on sexual dysfunctions and gynecomastia in male rheumatological patients treated with Methotrexate.

A systematic review was performed according to preferred reporting items for systematic reviews and meta-analyses (Figure 1) [21,22]. The systematic review protocol was registered with PROSPERO. Studies (1 January 1995 to 31 May 2022) were identified by highly sensitive searches of electronic databases (Medline, Embase, Cochrane Library databases). The literature search was performed in April and May 2022. The search was complemented by additional sources including the reference lists of included studies. The protocol was submitted to PROSPERO (CRD42022358275).

Key terms included: ((“sexual dysfunction” OR “erectile dysfunction” OR “impotence”) AND (“methotrexate” OR “MTX”)) and ((“gynecomastia” OR “male breast”) AND (“methotrexate” OR “MTX”)). Reference lists were also screened for additional studies according to the previous keywords.

### 2.2. Selection Criteria

Two authors (a senior and a junior urologist resident) reviewed the records separately and individually selected relevant publications, with any discrepancies resolved by a third senior author (an associate professor). An initial screening of titles and abstracts was performed to determine which papers could meet the inclusion criteria. Subsequently, the full-text articles underwent a more exhaustive assessment. PICOS (Population, Intervention, Comparison, Outcome, Study design) criteria were used to assess the eligibility of studies [22,23]. PICOS criteria were set as follows: (P) Male patients with rheumatic diseases; (I) MTX; (C) None; (O) Sexual dysfunction (SD) or gynecomastia; (S) Prospective and retrospective primary studies; including case series case reports, and abstract. Letters to the editor, editorial comments, reviews, and original articles without primary data were excluded. Ethical approval and patient consent were not required for the present study.

### 2.3. Data Collection

The following data were extracted: first author, year of publication, country of study, study design, study period, number of patients, follow-up, age, comorbidities, rheumatic disease, treatment before MTX, dosage of MTX, clinical presentation of SD/gynecomastia, and time to onset after starting MTX (Table 1).

### 2.4. Quality Assessment

The methodological quality of case reports and case series was performed according to Murad et al. [27] (Table 2). This tool is used for CR and CS and the result is to sum the scores of the eight binary responses into an aggregate score [27]. The evaluation of the level of evidence was performed according to the Oxford Center for Evidence-Based Medicine 2011 [28]. The Grading of Recommendations, Assessment, Development, and Evaluations (GRADE) system was used to evaluate the quality of the evidence [28,29].

### 2.5. Data Analysis and Synthesis

As a relatively low number of relevant papers with high heterogeneity in methodology were expected, quantitative data were reported as found in the original studies, according to a previous and similar methodology [2,20,29,30]. Percentages and medians were used to pool the extracted data. The main findings of the included papers were also summarized qualitatively. A meta-analysis could not be performed, since such an analysis may have been highly biased by the heterogeneity of the studies included. Specifically, the highest heterogeneity was recorded for clinical patient characteristics.

## 3. Results

The search strategy revealed a total of 58 results. The further assessment of articles, based on the full text, led to the exclusion of 39 papers. A total of seven papers (seven case reports), involving a total of eleven patients (min one, max three), were included in the final analysis (Figure 1). According to the GRADE system, the quality of evidence was “moderate”.

### 3.1. Patients’ Characteristics

Study characteristics and patients’ clinic-demographic profile were reported in Table 1. The age of the patients ranged from 19 to 68 years (median: 50.9 years), not being available in three papers. Rheumatoid arthritis (RA) was the most frequent rheumatic disease reported (n = 8, 72.7%). Two patients were affected by psoriatic arthritis (18.1%), and one by Adult Still’s disease (9.0%). No patients reported ED and/or gynecomastia before using MTX.

### 3.2. Methotrexate Administration

Follow-up of patients varied between 1 and 24 months (median: 9 months). A combination therapy was administrated before MTX in 11 patients (100%). The starting dose (mg) of MTX was 12.5 (n = 2, 18.1%), 10 (n = 2, 18.1%), 7.5 (n = 4, 36.3%), 5 (n = 1, 9.0%), and 4 (n = 2, 18.1%). The median starting dose was 10.81 mg. During the therapeutic period, six (6.7%) patients changed dosage. The final dose (mg) of MTX was 15 (n = 1, 9.0%), 12.5 (n = 2, 18.1%), 10 (n = 2, 18.1%), 8 (n = 1, 9.0%), 7.5 (n = 3, 27.2%), 6 (n = 1, 9.0%), and 5 (n = 1, 1.1%). The median final dose was 12,44 mg.

### 3.3. Adverse Events After Methotrexate Therapy

The time to onset of SD and gynecomastia after MTX ranged from 2 to 104 weeks (median: 22.7 weeks). ED (n = 5, 45.4%) and only gynecomastia (n = 3, 27.2%) were the most common forms of presentation. Other patients complained of Peyronie’s disease (PD) (n = 2, 18.1%), ED plus anejaculation (n = 2, 18.1%), and ED plus gynecomastia (n = 2, 18.1%).

## 4. Discussion

SD and gynecomastia were associated with rheumatic diseases, but limited evidence is currently available on the topic [21,26,31,32]. Rheumatic diseases certainly play a key role in the onset of SD, especially ED and loss of libido, both through direct physical effects and the resulting psychological repercussions [33]. Indeed, Boussaid et al. properly highlighted the unmet need of official guidelines to address SD in rheumatological patients, due to the increased incidence of hormone imbalance and impaired sperm quality of this cohort of patients [21]. The link between rheumatic diseases and gynecomastia was less immediate, but it probably derives from the association of these pathologies with other conditions that cause hormonal alterations [34]. We recorded that RA was the most frequent rheumatic disease associated with ED, loss of libido, and gynecomastia [33]. The explanation was due to the prevalence of RA within the rheumatological patients in comparison to the other autoimmune diseases reported. However, MTX is the most common immunosuppressant, representing the first-line treatment of RA and other rheumatological diseases. As a result, the magnitude of this drug on the occurrence and the potential link with SD and gynecomastia is worthy of exploration. Due to the paucity of the data reported in the literature, the association between comorbidities and the occurrence of rheumatological diseases as well as the impact of comorbidities (such as diabetes, hypertension, and metabolic syndrome) were not well known, and they should be addressed in controlled clinical trials or prospective studies with larger sample sizes and a better control of clinical and laboratory variables.

From our analysis, we recognized that these side effects occurred between 2 and 102 weeks after MTX introduction. Treatment discontinuation was the most reported management, and normality was recovered between 2 and 12 weeks after stopping the drug. We hypothesized that SD and gynecomastia in subjects using MTX was not dose-related or influenced by previous medical treatments. Indeed, both the starting and final dose ranged widely between 4 and 12.5 mg/Kg and previous therapies were the most disparate. Although the exact mechanism underlying SD and gynecomastia in patients taking MTX remains still unclear, several hypotheses have been suggested. First of all, MTX inhibits the enzyme DHFR, which catalyzes the conversion of dihydrofolate into tetrahydrofolate, the active form of folic acid (FA) [10]. Recently, FA has been recognized as an important molecule capable of increasing the bioavailability of nitric oxide (NO), stabilizing the NO synthase (NOS) [35]. Moreover, FA increases the bioavailability of tetrahydrobiopterin (BH4), an essential cofactor for dimerization of pteridine requiring monooxygenase enzymes, such as NOS, and consequently NO production [36]. Finally, FA upregulates the activity of DHFR in the biopterin recycling pathway and enhances the binding affinity of BH4 for endothelial NOS (eNOS), ensuring an improved redox state and optimal endothelial NO-dependent vasodilation [36]. MTX inhibits NO production through the inhibition of constitutive and/or inducible NOS by a DHFR-dependent pathway and reducing mRNA transcription of NOS genes [3,37]. MTX could determine gynecomastia with unclear mechanisms that may include changes in FA levels [25,38]. Indeed, FA levels may play a key-role in sexual hormonal pathways or may be a regulator of aromatases and androgenic carriers. As previously reported in the literature, MTX can induce oligospermia, decreased serum testosterone, and elevated serum follicle-stimulating hormone (FSH) levels [10]. Alternatively, MTX can favor oligospermia and elevated serum FSH levels in the presence of normal serum testosterone and luteinizing hormone levels [25]. Therefore, MTX-related gynecomastia may be caused firstly by a testicular failure and secondly by a subtle increase in the serum estrogen/androgen ratio. Furthermore, an increased aromatization of androgen, an enhanced synthesis of androgenic carriers, or an increased bioavailability of estrogen resulting from the removal of estrogen from its carrier proteins may also lead to MTX-related gynecomastia [25]. Finally, MTX can promote gynecomastia by blocking the activity of IL-1, which has been shown to influence pituitary hormone secretion, including prolactin [39,40]. Fukushi et al. highlighted that folate supplementation at a dosage of 5 mg/day in patients with gynecomastia gradually improved breast tenderness until the symptoms’ resolution within 2 months [25]. It is noteworthy to acknowledge that these patients can be helped by using folate supplementation upfront. Moreover, several advances in the management of these patients should be promoted. First, regular medical consultations should be scheduled with rheumatologists, endocrinologists, and sexual health experts in order to promptly diagnose and manage these events [41]. Second, correct information on the sexual MTX-adverse events should be disseminated, reducing the misdiagnosis of such rare, albeit real, conditions. We also recorded cases of PD and anejaculation among patients taking MTX. This association, if not causal, certainly deserves further investigation to generate plausible pathophysiological hypotheses.

Interestingly, none of those studies enlightened understanding of the psychological imbalance of the patients affected by gynecomastia or ED. Specifically, both conditions share the impairing of social functioning, mental health, and self-esteem [42,43]. Li et al., for instance, reported that 94.8% of patients with gynecomastia reported psychological stress [44]. Due to the higher mental health burden associated with these conditions, it may be necessary to carry out preoperative counselling those patients with a psychological interview. Indeed, the psychological evaluation may either help to prevent or diagnose underlying conditions that may also affect the psychological balance in gynecomastia/ED patients.

To the best of our knowledge, this is the first systematic review on SD and gynecomastia in patients with rheumatic diseases treated with MTX. Consequently, our article contributed to improving knowledge on the topic and could pave the way for a new line of research. The rigorous methodology is a relevant strength of our study. However, our findings and hypothesis should be considered with caution due to several limitations. The main issues are the poor methodological quality and the low level of evidence of available data. Indeed, only case series and case reports were deemed eligible for the data extraction. Given the paucity, heterogeneity, and poor quality of the data, as suggested by previous recent analyses [21], we refrained from conducting a meta-analysis, recognizing its potential unreliability in such circumstances. Nevertheless, it has served as an essential initial step by offering a descriptive framing of the topic and generating hypotheses for future research. Specifically, the dose–response relationship of MTX and its impact on sexual hormones should be investigated in wider sample size studies. Moreover, a multidisciplinary team (including urologists, rheumatologists, endocrinologist, and sexual health experts) may be organized to manage patients with MTX adverse events.

Additionally, there is a clear imperative for well-designed prospective studies and clinical trials to provide more robust evidence. Future research should aim to overcome the limitations identified in our review, such as the reliance on case reports and the methodological shortcomings of existing studies. Additionally, exploring the underlying mechanisms and potential risk factors associated with SD and gynecomastia in patients undergoing MTX treatment would be a valuable avenue for investigation. It is noteworthy to acknowledge that those patients may be prone to a higher mental health burden, due to their sexual impairment. Thus, it is crucial and appropriate that patients receive counselling that should be focused on these rare, albeit real, adverse effects. Moreover, new established protocols of folate supplementation could be administered to avoid sexual dysfunction. Collaboration among multidisciplinary teams, including rheumatologists, endocrinologists, and sexual health experts, could enrich the depth of future investigations. This collaborative approach may facilitate a comprehensive understanding of the complex interplay between rheumatic diseases, MTX, and the observed adverse effects. It could also anticipate the detection of the potential life-threatening consequences of MTX administration.

## 5. Conclusions

SD and gynecomastia appear to be associated prevalently with MTX treatment of RA. Unfortunately, our findings should be considered with caution due to several limitations, such as the low level of evidence of available data. Despite those limitations, the current work has served as an essential initial step by offering a descriptive framing of the topic and generating hypotheses for future research. Specifically, the dose–response relationship of MTX and its impact on sexual hormones should be investigated in wider sample size studies. Moreover, a multidisciplinary team (including urologists, rheumatologists, endocrinologist, and sexual health experts) may be organized to manage patients with MTX adverse events. Last but not the least, physicians should be aware of the possible side effects and adequately inform the patient before starting treatment. Future prospective controlled studies and controlled clinical trials with a large sample size and long follow-up are needed to confirm this association, investigate its pathophysiological basis, assess the safest dosages, evaluate the most appropriate management, and provide clear recommendations.

## Figures and Tables

**Table 1:** Study characteristics and patients ‘clinic demographic data.

Authors	Type of Study and Numerosity	Age (Median; IQR)	Comorbities	Disease	Starting Dosage (mg/Weekl y)	Ending Dosage (mg/Weekl y)	Sexual Dysfunction	Time to Presentation (Weeks)	Treatments	Time to Recovery (Weeks)	Follow Up (mo.)
Del Paine, 1983 [19]	CR, 1	19	NA	Adult still’s disease, 1	7.5, 1	7.5, 1	G, 1	2	TI, 1	1, 1	1, 1
Blackburn, 1989 [9]	CR, 3	52.33 (47–62)	NA, 3	AR, 3	12.5, 1 7.5, 1 5, 1	12.5, 1 12.5, 1 5, 1	ED + A, 2 ED,1	6, 1 NA, 2	TI,1 DR,1 0, 1	2, 1 NA,1 NR, 1	2, 1 NA, 2
Phelan, 1992 [24]	CR, 2	53 (48–58)	NA, 2	AR, 2	10,2	10, 1 15, 1	PD, 2	20, 1 12, 1	TI, 2	12, 2	10, 1 9, 1
Aguirre, 2002 [17]	CR, 2	36 (30–42)	0, 1 NA,1	PsAF, 2	7.5, 2	7.5, 2	ED + G, 2	52, 1 104, 1	TI, 2	NA, 2	12, 1 24, 1
Abe, 2007 [18]	CR, 1	65	NA	AR, 1	4, 1	6, 1	G, 1	12, 1	TI, 1	12, 1	7, 1
Fukushi, 2009 [25]	CR, 1	62	NA	AR, 1	4, 1	8, 1	G,1	32, 1	FS, 1	8, 1	10, 1
Shaikh, 2016 [26]	CR, 1	51	NA	AR, 1	10, 1	10, 1	G, 1	10 (8–12), 1	FS + TI, 1	8, 1	5, 1

**Table 2:** Methodological quality of case reports and case series according to Murad et al. [27].

Domains	Selection	Ascertainment		Causality				Reporting
Leading Explanatory Questions	1	2	3	4	5	6	7	8
Del Paine, 1983 [19]	no	yes	no	yes	no	no	yes	yes
Blackburn,1989 [9]	yes	yes	yes	no	yes	Yes	yes	yes
Phelan, 1992 [24]	no	yes	yes	no	no	no	yes	yes
Aguirre, 2002 [17]	no	Yes	No	no	no	no	yes	yes
Abe, 2007 [18]	no	yes	yes	yes	no	yes	yes	yes
Fukushi, 2009 [25]	no	yes	yes	yes	no	no	yes	yes
Shaikh, 2016 [26]	no	yes	yes	yes	no	no	yes	yes

Leading explanatory questions: 1. Does the patient(s) represent(s) the whole experience of the investigator (center) or is the selection method unclear to the extent that other patients with similar presentation may not have been reported? 2. Was the exposure adequately ascertained? 3. Was the outcome adequately ascertained? 4. Were other alternative causes that may explain the observation ruled out? 5. Was there a challenge/rechallenge phenomenon? 6. Was there a dose–response effect? 7. Was follow-up long enough for outcomes to occur? 8. Is the case(s) described with sufficient detail to allow other investigators to replicate the research or to allow practitioners make inferences related to their own practice?

## Data Availability

The specific datasets generated during and/or analyzed during the current study are available from the corresponding author on reasonable request.
